# Reduced Serum PD-L1 and Markers of Inflammation in Response to Alternate Day Fasting With a Low-Carbohydrate Intervention: A Secondary Analysis of a Single-Arm Trial

**DOI:** 10.1016/j.cdnut.2025.104566

**Published:** 2025-02-14

**Authors:** Rand T Akasheh, Giamila Fantuzzi, Krista A Varady, Ting-Yuan D Cheng, Faiza Kalam

**Affiliations:** 1 Division of Cancer Prevention and Control, Department of Internal Medicine, The Ohio State University Comprehensive Cancer Center – The James Cancer Hospital and Solove Research Institute, College of Medicine, The Ohio State University Wexner Medical Center, Columbus, OH, USA; 2 Department of Kinesiology and Nutrition, College of Applied Health Sciences, University of Illinois Chicago, Chicago, IL, USA

**Keywords:** Intermittent fasting, alternate day fasting, low-carbohydrate diet, obesity, weight loss, inflammation, cytokines, chemokines, PD-L1

## Abstract

This secondary analysis aimed to examine the effect of a single-arm alternate day fasting intervention with a 30% low-carbohydrate diet on biomarkers of inflammation and immune activation in adults with obesity. A 12-week weight-loss period was followed by a 12-week weight maintenance period. Anthropometrics and blood samples were collected at baseline and weeks 12 and 24. Multiplex assay was used to measure serum biomarkers including programmed death ligand 1 (PD-L1), interleukin 8 (IL-8), IL-1 receptor antagonist (IL-1ra), chemokine ligand (CCL)2, CCL4, interferon gamma (IFnγ), IFNγ–induced protein 10 (IP-10), and cluster of differentiation 40 ligand (CD40-L). In 28 participants, body weight and fat mass decreased during the weight-loss period but stabilized during the weight maintenance period. Serum PD-L1 decreased from baseline to week 12 (*P* = 0.005) but not at week 24. Moreover, IL-1ra and CCL4 concentrations decreased from baseline to week 24 (*P* < 0.001 and *P* < 0.008, respectively). Changes were not significant for in CCL2, IL-8, CD40-L, IFNγ, or IP-10. In conclusion, alternate day fasting–low carbohydrate modulates circulating immune biomarkers, which may be relevant to diabetes, cancer, and autoimmunity.

This trial was registered at clinicaltrials.gov as NCT03528317 (https://www.ncbi.nlm.nih.gov/pmc/articles/PMC6934424/).

## Introduction

Alternate day fasting (ADF) and low-carbohydrate (LC) diets are recognized for their benefits in weight management and metabolic health [[1], [2], [3]]. ADF is a type of intermittent fasting that involves alternating between days of severe caloric restriction and ad libitum eating [1,4], although LC diets restrict carbohydrate intake in favor of higher proportions of protein and fat [5]. Although these dietary patterns have been extensively studied for their effects on weight loss, cardiometabolic health, and hormones [[6], [7], [8], [9], [10]], their impact on immune response biomarkers remains relatively underinvestigated.

Leukocyte activation is a complex process that is regulated by various molecules, cytokines, and chemokines [11]. Obesity alters immune functions, and dietary patterns can influence the immune system through various mechanisms, including modulation of inflammatory pathways, leukocyte polarization, and cytokine production [[12], [13], [14]]. However, the effects of ADF-LC diet on many immune biomarkers are largely unknown. For example, although programmed death ligand (PD-L) 1, chemokine ligand (CCL)2, CCL4, interleukin 8 (IL-8), IL-1 receptor antagonist (IL-1ra), interferon gamma (IFNγ), cluster of differentiation 40 ligand (CD40-L), and IFNγ–induced protein (IP-10) are all upregulated in obesity, the effects of weight loss and dietary interventions on these biomarkers have been understudied. These molecules and cytokines are relevant to many diseases related to obesity including diabetes, cancer, and autoimmunity. For instance, immune checkpoint molecules like the inhibitory PD-L1 and the stimulatory CD40 are expressed on normal and malignant cells to enable immune tolerance/evasion or activation, respectively, playing key roles in anti-cancer immunity and autoimmune diseases [15,16]. The cytokine IL-1ra antagonizes the inflammatory effects of IL-1β, halting a key pathway in the development of type 2 diabetes [17]. The chemokines CCL2 and CCL4 recruit leukocytes to the site of injury or cancer [18,19], although IFNγ and its inducible IP-10 activate lymphocytes against infections or tumors [20]. Therefore, studying the effects of ADF-LC on these immune biomarkers will provide insights and generate hypotheses to better understand the role of obesity-induced inflammation and its relation to chronic diseases.

This study was a secondary analysis of a single-arm weight loss clinical trial for adults with obesity, which aimed to investigate the effects of ADF-LC on circulating immune response biomarkers that are modulated by obesity including PD-L1, CCL2, CCL4, IL-8, IL-1ra, IFNγ, CD40-L, and IP-10.

## Methods

### Participant recruitment

The protocol was approved by the Office for the Protection of Research Subjects of the University of Illinois Chicago, with an institutional review board approval number of 2017-1363. Flyers were distributed across the university campus. Eligible participants met the following criteria: BMI of 30.0–49.9 kg/m^2^, age of 18–65 years, pre- or post-menopausal (for women), lightly active (<3 h/wk of light exercise), weight stable for 3 months, no history of diabetes or cardiovascular disease, nonsmokers, not nightshift workers, and not on weight-loss medications. Over a period of 8 months, 31 participants were recruited and provided written informed consent.

### Experimental design

A secondary analysis of a 6-month single-arm intervention entailing a 12-wk weight-loss period followed by a 12-wk weight maintenance period, to a total of 24 wk. Details on the protocol and the primary findings have been published [21]. For this analysis, sera were only available for 28 of 31 participants.

### ADF with a LC intervention

Meal replacements (Optifast HP Shake Mix; Nestle) were provided to help participants adhere to calorie and macronutrient targets. Each shake in the form of powder sachet contained 26 g protein, 10 g carbohydrates, 6 g fat, and 200 kcal. A macronutrients distribution of 30% carbohydrates, 35% protein, and 35% fat was used in this intervention.

During the weight-loss period (weeks 0–12), participants consumed 3 meal replacements totaling 600 kcal, with only water, black coffee, and tea allowed at any time on fast days, although they consumed 5 meal replacements totaling 1000 kcal in addition to ad libitum eating on feast days. During the weight maintenance period (weeks 13–24), participants continued with 3 meal replacements totaling 600 kcal on fast days and 3 meal replacements totaling 600 kcal with ad libitum eating on feast days. Participants were allowed to eat ad libitum on feast days only after consuming their meal replacements.

### Anthropometric and dietary assessments

Height was measured at baseline using a stadiometer. Body weight was measured biweekly using a digital scale. Body composition was assessed using dual-energy X-ray absorptiometry at baseline and at the end of weeks 12 and 24.

A dietitian provided biweekly counseling on selecting LC foods to meet macronutrient targets and reviewed the daily shake adherence log. Percent adherence was calculated as follows: (No. of shakes consumed/No. of shakes distributed) × 100%. Participants also completed 7-d food records at baseline and weeks 12 and 24. Nutrient intakes were calculated using Nutritionist Pro™ (Axxya Systems LLC). To monitor the intake of meal replacements, participants were asked to return any shakes that were not consumed to the research facility and were promised to receive them back at the end of the trial.

### Measurement of circulating biomarkers

Blood samples were collected from participants at baseline, and at the end of weeks 12 and 24 after an overnight fast following a feast day and processed for sera. Luminex assay (Biotechne) was used to measure PD-L1, CD40-L, IFNγ, CCL2, CLL4, IP10, IL-8, and IL-1 receptor antagonist (IL-1ra) in duplicates. Positive and negative controls were assayed with each kit to assure data quality and accuracy.

### Statistical analysis

Data were analyzed using JAMOVI (version 2.3.28). Normality was assessed with Shapiro–Wilk test. Outliers were identified and excluded based on Grubb test and clinical judgment. Pearson correlation coefficients were calculated to evaluate the association between serum biomarkers and body size and composition variables at baseline. Repeated-measures ANOVA was used to compare the changes in biomarkers at week 12 and 24 with those at baseline. A *P* < 0.05 implied statistical significance.

## Results

Study participants had a mean age of 48.6 y and were predominantly females and African Americans. Following the ADF-LC intervention, body weight decreased with mean weight-loss percentages of −5.26% and −5.88% (*P* < 0.001) at weeks 12 and 24, respectively (*P* < 0.001) (Table 1). BMI, waist circumference, and body fat percentage decreased (all *P* < 0.001), whereas lean mass and lean mass index showed a significant reduction by week 12 (*P* < 0.001) and a slight increase by week 24 compared with that at week 12 (*P* = 0.013). No significant changes were observed in visceral fat mass (*P* = 0.097). Anthropometric changes paralleled reductions in energy intake in both feast and fasting days (Table 1).

**TABLE 1:** Baseline measures and anthropometric changes at weeks 12 and 24 of alternate day fasting plus low-carbohydrate intervention.

Variables	Baseline	Week 12	Week 24	*P*
n	28	28	28	—
Age (y)	48.6 (1.7)	—	—	—
Sex (female/male)	23/5	—	—	<0.001
Race/ethnicity				0.001
African American	16	—	—	—
Hispanic	8	—	—	—
Asian	3	—	—	—
White	1	—	—	—
Anthropometrics				—
Height (cm)	162 (1.7)	—	—	—
Body weight (kg)	100.1 (3.81)	95.0 (3.84)∗∗∗	94.3 (3.83)∗∗∗	<0.001
Weight loss %	0.0 (0.0)	−5.26 (0.58)∗∗∗	−5.88 (0.98)∗∗∗	<0.001
BMI (kg/m^2^)	37.9 (1.13)	35.9 (1.12)∗∗∗	35.7 (1.17)∗∗∗	<0.001
Lean mass (kg)	49.7 (1.71)	48.5 (1.67)∗∗∗	49.3 (1.71)^#^	<0.001
Lean mass index (kg/m^2^)	18.8 (0.37)	18.4 (0.38)∗∗∗	18.7 (0.4)^#^	<0.001
Fat mass (kg)	45.6 (2.64)	41.7 (2.57)∗∗∗	41.0 (2.70)∗∗∗	<0.001
Body fat percentage (%)	47.0 (1.22)	46.0 (1.38)∗	44.7 (1.27)∗∗∗^,###^	<0.001
Visceral fat mass (kg)	1.34 (0.12)	1.21 (0.13)	1.21 (0.13)	0.097
Waist circumference (cm)	105.6 (2.07)	97.7 (2.10)∗∗∗	95.9 (2.05)∗∗∗^,#^	<0.001
Energy intake (kcal)				
Feast days	2366 (245)	1627 (86)∗∗	1621 (112)∗∗	0.001
Fast days	2366 (245)	1188 (117)∗∗∗	1293 (100)∗∗∗	<0.001

The intervention entailed a 12-wk weight-loss phase ending at week 12 and then another 12-wk weight maintenance phase ending at week 24. Data are presented as means (SEM) for age and anthropometric variables. *P* value reported from repeated-measures ANOVA with time (baseline, week 12, and week 24) as the within-subject factor. ∗*P* < 0.05 and ∗∗∗*P* < 0.001 for week 24 or 12 compared with baseline based on multicomparison test. ∗*P* < 0.05 and ∗∗∗*P* < 0.001 for week 24 or 12 compared with baseline based on multicomparison test. ^#^*P* < 0.05 and ^###^*P* < 0.001 for week 24 compared with week 12 based on multicomparison test. Height *P* value was based on independent sample *t* test comparing treatment with control groups. Body composition components (fat mass, lean mass, and visceral fat mass) were measured using dual-energy X-ray absorptiometry.

**FIGURE 1:**
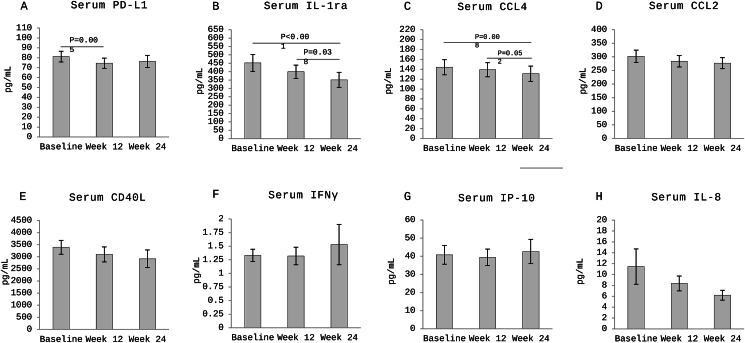
Changes in serum immune biomarkers in response to alternate day fasting combined with low-carbohydrate diet intervention. Serum concentrations are presented as mean ± standard error. *P* value of <0.05 indicates statistical significance. Serum concentrations of IP10, IL-1ra, IFNγ, CCL4, and IL8 are log-transformed. The intervention entailed a 12-wk weight-loss phase ending at week 12 and then another 12-wk weight maintenance phase ending at week 24. PD-L1, programmed death ligand 1; IL-1ra, IL-1 receptor antagonist; CCL, chemokine ligand; CD40L, cluster of differentiation-40 ligand; IFN, interferon; IP-10: interferon γ–induced protein.

At baseline, only waist circumference among anthropometric indicators positively correlated with baseline serum IL-1ra (*r* = 0.451; *P* < 0.05) but not with any of the other biomarkers (Supplemental Table 1). In response to the intervention, serum PD-L1 concentrations significantly decreased from baseline (81.2 ± 5.43 pg/mL) to week 12 (74.5 ± 5.21 pg/mL; *P* = 0.005). This decrease was maintained through week 24 (76.3 ± 6.07 pg/mL) but was not statistically significant (*P* = 0.203). No significant change was observed from week 12 to 24 (Figure 1A). Furthermore, there was a significant reduction in serum IL-1ra concentrations from baseline (452 ± 50.1 pg/mL) and week 12 (399 ± 39.9 pg/mL) to week 24 (351 ± 44.6 pg/mL; *P* < 0.001 and *P* < 0.038, respectively) (Figure 1B). Serum CCL4 concentrations also significantly decreased from baseline (144 ± 15.3 pg/mL) to week 24 (131 ± 15.4 pg/mL; *P* = 0.014) but nonsignificantly to week 12 (139 ± 14.3 pg/mL; *P* = 0.091) (Figure 1C). Furthermore, a trend toward decreased monocyte chemotactic protein 1 and CD40-L concentrations was observed over time (all *P* > 0.05) (Figure 1D and E). No significant changes were observed in serum IFNγ, IL-8, and IP-10 concentrations over the 24-wk period (Figure 1F–H). Although weight-loss percentage at week 12 did not significantly correlate with any immune biomarker changes, the change in fat-free mass index significantly and negatively correlated with the changes in serum PD-L1 (*r* = −0.561; *P* < 0.01), IL-1ra (*r* = −0.489; *P* < 0.01), and IFNγ (*r* = −0.42; *P* < 0.05). Moreover, the changes in body fat percentage and waist circumference did not correlate with the changes observed in immune biomarkers, but the changes in visceral fat mass positively correlated with the changes in CD40-L (*r* = 0.526; *P* < 0.01) and IL-1ra (*r* = 0.478; *P* < 0.05) (Supplemental Table 2). However, no significant correlations were observed at week 24 between changes in anthropometric indicators and changes in immune biomarkers (Supplemental Table 3).

## Discussion

The aim of this study was to investigate the effects of ADF combined with LC diet on serum immune biomarkers relevant to cancer immunotherapy over a 24-wk period. The main findings indicate that the ADF-LC intervention decreased serum PD-L1, IL-1ra, and CCL4 concentrations, with these changes being correlated with changes in lean mass but not weight loss or fat loss. These findings may have implications for many diseases such as type 2 diabetes, autoimmune conditions, and cancer. Thus, this discussion will focus on the translational potential of these findings in these diseases.

Caloric restriction and intermittent fasting modulate immune function potentially enhance antitumor immunity [22,23]. For example, a cyclic fasting mimicking diet reduces immunosuppressive leukocytes and enhances antitumor Th1/cytotoxic responses and IFNγ signaling in tumor leukocytes [24]. Additionally, short-term starvation enhances the efficacy of PD-1/PD-L1 blockade in mice [25]. However, to our knowledge, the effects of intermittent fasting and the concurrent weight loss on serum markers of immune activation including PD-L1 have not been reported. In this study, serum PD-L1 decreased by week 12 compared with that at baseline, suggesting a potential immunomodulatory effect of the ADF-LC intervention. The immune checkpoint molecule PD-L1 binds to its receptor PD-1 on T-lymphocytes, suppressing immune activity and allowing cancer cells to evade destruction by cytotoxic T-lymphocytes. Notably, PD-L1 is upregulated in visceral adipose tissue of individuals with obesity [26]. In patients with triple-negative breast cancer, BMI positively but nonsignificantly correlates with tumor PD-L1 expression (*R* = 0.249; *P* = 0.055) [27]. Furthermore, obesity may be associated with better immunotherapy responses but more adverse events [26,28]. Thus, the reduced PD-L1 concentrations along with an average weight loss of 5.88% body weight achieved in this study aligns with previous reports suggesting that body weight change may affect PD-L1 expression and, for the first time, presents evidence that dietary interventions can alter PD-L1 expression. However, changes in PD-L1 along with IL-1ra and IFNγ in this study correlated with changes in fat-free mass rather than weight or fat loss, implying that muscle mass changes can mediate changes in immune function. It remains unclear whether circulating PD-L1 concentrations parallel its adipose tissue or tumor expression. The observed reduction in serum PD-L1 suggests that the ADF-LC intervention could enhance T cell–mediated tumor suppression, potentially improving immune checkpoint inhibitor efficacy in cancer treatment. However, more research is necessary to directly correlate these findings with clinical outcomes in cancer patients. From another view, PD1/PD-L1 signaling is also implicated in autoimmune diseases, whereby inhibiting PD1 or PD-L1 in mice accelerates the development of type 1 diabetes, although upregulating PD-L1 protects against it [16]. Therefore, the clinical significance of reduced PD-L1 in response to ADF-LC remains unclear and is probably context dependent.

The low-grade chronic inflammation of obesity is a driving mechanism of many chronic diseases. On the contrary, fasting and caloric restriction can reduce inflammation and alter cytokine concentrations [4,29]. A meta-analysis of randomized clinical trials reported that intermittent fasting significantly reduces circulating high-sensitivity C-reactive protein but not TNF or IL-6 concentrations [29]. In this study, serum IL-1ra concentrations decreased by week 24 compared with that at baseline and week 12 in response to ADF-LC. Although IL-1 family of proteins bind to the receptor IL-1 receptor to induce inflammation, IL-1ra is a nonactivating ligand of the same receptor that prevents the induction of this inflammatory pathway [30]. Importantly, IL-1ra is upregulated in obesity [17]. Thus, its downregulation with weight loss induced by ADF-LC might suggest overall alleviation in obesity-induced inflammation and the compensatory anti-inflammatory response. However, in future studies, these findings must be observed and interpreted in parallel to changes in IL-1β, a cytokine increasingly recognized as a central player in the development of insulin resistance and type 2 diabetes and that is antagonized by IL-1ra [17]. Additionally, although inflammation drives tumor development and progression [31], IL-1ra inhibits tumor growth and metastasis [30]. Thus, the clinical impact of ADF-LC in the context of diabetes and cancer should be explored.

Elevated CCL4 and CCL2 expression were observed in subcutaneous adipose tissue cells isolated from patients with obesity compared with those in patients with normal body weight [32]. Also known as macrophage inflammatory protein-1β, CCL4 is like other chemokines involved in the recruitment of leukocytes to the site of inflammation [33]. Thus, the reduction in serum CCL4 observed at weeks 12 and 24 of ADF-LC suggests potential anti-inflammatory benefits of this diet.

The decreases in serum CCL2 and CD40-L concentrations in response to ADF-LC were not statistically significant. However, longer or more intensive dietary interventions might produce more pronounced effects on these biomarkers. The chemokine CCL2, also named monocyte chemotactic protein, recruits monocytes to sites of inflammation. Although CCL2 has strong proinflammatory effects, the activation of CCL2 pathway in CCR2-expressing macrophages and regulatory T cells exerts potent immunosuppressive effects in the tumor microenvironment, and hence, targeting the CLL2/CCR2 axis as a cancer immunotherapy method is underinvestigation [34]. Furthermore, the costimulatory protein CD40-L, expressed on T cells, binds to CD40 receptors on B cells inducing their clonal expansion and is associated with insulin resistance in people with obesity [35]. The CD40-L/CD40 axis is also a target of immune checkpoint inhibitor drugs [15]. Therefore, expanding our understanding of the effects of ADF-LC and other dietary interventions on this pathway is crucial.

The ADF-LC intervention did not exert substantial changes to serum IFNγ, IL-8, and IP-10 concentrations. The proinflammatory cytokine IFNγ is critical for antiviral and antitumor immunity [36]. Both IFNγ and IL-8 are upregulated in obesity compared with those at normal weight [37] and downregulated at 6 and 12 months following surgical weight loss [37]. Moreover, IP-10 is upregulated in morbid obesity compared with normal weight [20]. It is possible that the intensity and length of the ADF-LC intervention in this study was insufficient to affect these biomarkers or that ADF-LC modulates certain pathways of inflammation and immunity but not others, highlighting the complexity of modulating the immune system through dietary means. Overall, the findings of this study underscore the potential of dietary interventions like ADF-LC in modulating inflammatory and immune biomarkers, which could have implications in the prevention and treatment of many diseases. However, further studies are necessary to fully understand the mechanisms involved and to establish protocols that convey clinical benefits.

To our knowledge, this study is the first to explore the combined effects of LC with ADF on immune response biomarkers over 24 wk. It is also the first to investigate the impact of ADF-LC on serum immune biomarkers relevant to obesity, autoimmunity, and cancer immunology, particularly PD-L1, offering insights into designing dietary interventions for cancer prevention, autoimmune disease management, or immune checkpoint inhibition enhancement in cancer treatment. However, serum concentrations of PD-L1 and other biomarkers do not necessarily reflect their expression levels in healthy or neoplastic tissue. Moreover, studying immune biomarkers was not the primary aim of the original trial. In addition, PD-L1 changes did not correlate with weight loss, because the sample size is underpowered for this secondary analysis, the ADF-LC dietary content played a greater role in serum change, or the serum changes depended on body composition changes. Moreover, the study only included otherwise healthy people with obesity, which limits the applicability of the findings to people with diabetes, autoimmunity, or cancer. Moreover, the lack of a control group in this study makes it unclear whether the observed effects can be attributed to the ADF, the LC, the energy restriction aspects of the study, or a combination of all. Participants underconsumed energy on feast ad libitum days but overconsumed energy on fasting days, either implying inaccurate food intake reporting or limited adherence to the prescribed diet. Therefore, these findings must be confirmed in future studies with larger, more diverse populations, focusing on possible implications in obesity and related diseases.

In conclusion, this study provides novel insights into the potential immunomodulatory and anti-inflammatory effects of a 24-wk ADF-LC intervention. The significant reductions in serum concentrations of PD-L1, IL-1ra, and CCL4 suggest potential benefits of the intervention for obesity, with implications in many diseases such as diabetes, cancer, and autoimmune conditions. Therefore, future studies may investigate the clinical relevance of these findings.

## Author contributions

The authors’ responsibilities were as follows – RTA: wrote the manuscript and analyzed and interpreted the data; T-YDC: designed this study and had the primary responsibility of the final content; FK: conducted the original trial; GF: provided critical input in study design, interpreted the data, and edited the manuscript; KAV: designed the original trial; and all authors: read and approved the final manuscript.

## Data availability

Data described in the manuscript, code book, and analytic code will be made available upon request pending application and approval.

## Funding

The original trial was funded by Nestle Health Science. The work presented in this manuscript was funded by The Ohio State University Comprehensive Cancer Center – James. The funding institutions were not involved in designing the study; collecting, analyzing, and interpretation of the data; writing of the report; or the submission of the report for publication.

## Conflict of interest

KV reports financial support and Optifast HP Shake Mix were provided by Nestle Health Science. All other authors report no conflicts of interest.
