# Supplementary material for: Reduced Serum PD-L1 and Markers of Inflammation in Response to Alternate Day Fasting With a Low-Carbohydrate Intervention: A Secondary Analysis of a Single-Arm Trial
Source: Curr Dev Nutr. 2025 Feb 14;9(3):104566. doi: 10.1016/j.cdnut.2025.104566 (PMC11938049; doi:10.1016/j.cdnut.2025.104566)
Supplement: Multimedia component 1 [file mmc1.docx]

**Reduced serum PD-L1 and markers of inflammation in response to alternate day fasting with a low-carbohydrate intervention: A secondary analysis of a single-arm trial**

**Author: Rand Talal Akasheh**

**Supplementary Table 1: Correlation matrix of baseline anthropometric measures and serum biomarkers in a cohort of adults with obesity**

|  | BMI | FFM | FFMI | BF% | VFM | WC | PDL1 | CD40L | CCL2 | CCL4 | IL1ra | IFNγ | IP10 | IL8 |
| --- | --- | --- | --- | --- | --- | --- | --- | --- | --- | --- | --- | --- | --- | --- |
| BMI | — |  |  |  |  |  |  |  |  |  |  |  |  |  |
| FFM | **0.403*** | — |  |  |  |  |  |  |  |  |  |  |  |  |
| FFMI | **0.617***** | **0.793***** | — |  |  |  |  |  |  |  |  |  |  |  |
| BF% | **0.92***** | **0.458*** | **0.447*** | — |  |  |  |  |  |  |  |  |  |  |
| VFM | 0.221 | 0.364 | **0.435*** | 0.11 | — |  |  |  |  |  |  |  |  |  |
| WC | **0.707***** | **0.586**** | **0.564**** | **0.741***** | **0.619***** | — |  |  |  |  |  |  |  |  |
| PDL1 | 0.104 | 0.026 | 0.085 | 0.039 | 0.117 | 0.201 | — |  |  |  |  |  |  |  |
| CD40L | -0.099 | 0.063 | -0.005 | -0.103 | 0.275 | -0.065 | 0.203 | — |  |  |  |  |  |  |
| CCL2 | -0.266 | 0.088 | 0.058 | -0.295 | 0.203 | 0.015 | -0.19 | 0.315 | — |  |  |  |  |  |
| CCL4 | 0.021 | 0.057 | 0.067 | 0.032 | -0.127 | 0.162 | **0.515**** | 0.023 | -0.17 | — |  |  |  |  |
| IL1ra | 0.261 | 0.146 | 0.118 | 0.296 | 0.266 | **0.451*** | 0.371 | **0.407*** | 0.269 | 0.179 | — |  |  |  |
| IFNγ | 0.131 | -0.039 | 0.177 | -0.048 | 0.354 | 0.192 | 0.228 | 0.253 | 0.268 | 0.067 | 0.173 | — |  |  |
| IP10 | 0.025 | -0.143 | -0.161 | 0.048 | 0.094 | 0.179 | 0.042 | 0.083 | 0.211 | 0.019 | 0.249 | **0.475*** | — |  |
| IL8 | -0.29 | 0.007 | -0.089 | -0.258 | -0.048 | -0.134 | -0.113 | 0.244 | **0.439*** | **0.453*** | 0.198 | 0.149 | 0.069 | — |

Data are presented as Pearson’s correlation coefficient.

Statistical significance is indicated by * p<0.05, ** p<0.01, or *** p<0.001.

Serum concentrations of IP10, IL1ra, IFNγ, CCL4, and IL8 are log-transformed.

BMI: Body mass index; FFM: Fat free mass; FFMI: Fat free mass index; BF%: Body fat percentage; VFM: Visceral fat mass; WC: Waist circumference; PDL1: Programmed death ligand 1; IL1-ra: Interleukin-1 receptor antagonist; CCL2: Chemokine ligand 2; CCL4: Chemokine ligand 4; CD40L: Cluster of differentiation-40 ligand; IFNγ: Interferon gamma; IL8: Interleukin-8; IP-10: IFNγ-induced protein.

**Supplementary Table 2: Correlation matrix of changes in anthropometric measures and serum biomarkers on week 12 relative to baseline in response to alternate day fasting plus low carbohydrate diet intervention.**

|  | WL% | ΔBMI | ΔFFM | ΔFFMI | ΔBF% | ΔVFM | ΔWC | ΔPDL1 | ΔCD40L | ΔCCL2 | ΔCCL4 | ΔIL1ra | ΔIFNγ | ΔIP10 | ΔIL8 |
| --- | --- | --- | --- | --- | --- | --- | --- | --- | --- | --- | --- | --- | --- | --- | --- |
| WL% | — |  |  |  |  |  |  |  |  |  |  |  |  |  |  |
| ΔBMI | **0.915***** | — |  |  |  |  |  |  |  |  |  |  |  |  |  |
| ΔFFM | 0.243 | 0.229 | — |  |  |  |  |  |  |  |  |  |  |  |  |
| ΔFFMI | 0.248 | 0.233 | **0.993***** | — |  |  |  |  |  |  |  |  |  |  |  |
| ΔBF% | **0.732***** | **0.759***** | 0.15 | 0.122 | — |  |  |  |  |  |  |  |  |  |  |
| ΔVFM | 0.025 | 0.046 | -0.016 | -0.026 | 0.187 | — |  |  |  |  |  |  |  |  |  |
| ΔWC | 0.241 | 0.269 | 0.143 | 0.149 | 0.324 | 0.01 | — |  |  |  |  |  |  |  |  |
| ΔPDL1 | 0.134 | 0.156 | **-0.555**** | **-0.561**** | -0.005 | 0.087 | 0.255 | — |  |  |  |  |  |  |  |
| ΔCD40L | 0 | 0.001 | -0.304 | -0.305 | -0.02 | **0.526**** | 0.073 | **0.518**** | — |  |  |  |  |  |  |
| ΔCCL2 | 0.003 | -0.134 | -0.048 | -0.056 | -0.005 | 0.326 | -0.274 | 0.076 | -0.025 | — |  |  |  |  |  |
| ΔCCL4 | -0.105 | -0.099 | -0.29 | -0.322 | -0.019 | 0.377 | 0.14 | **0.516**** | **0.422*** | 0.235 | — |  |  |  |  |
| ΔIL1ra | -0.234 | -0.198 | **-0.474*** | **-0.489**** | -0.144 | **0.478*** | -0.203 | **0.392*** | **0.68***** | 0.306 | **0.441*** | — |  |  |  |
| ΔIFNγ | -0.183 | -0.294 | **-0.391*** | **-0.42*** | -0.26 | 0.142 | -0.144 | **0.481*** | 0.201 | 0.146 | 0.292 | 0.246 | — |  |  |
| ΔIP10 | 0.137 | 0.068 | -0.14 | -0.198 | 0.022 | -0.162 | -0.205 | 0.33 | -0.13 | 0.172 | 0.197 | 0.109 | **0.517**** | — |  |
| ΔIL8 | -0.283 | -0.265 | -0.22 | -0.232 | -0.258 | 0.307 | -0.202 | **0.404*** | **0.529**** | 0.228 | 0.379 | **0.645***** | 0.32 | 0.186 | — |

Data are presented as Pearson’s correlation coefficient.

Statistical significance is indicated by * p<0.05, ** p<0.01, or *** p<0.001.

Serum concentration of IP10, IL1ra, IFNγ, CCL4, and IL8 are log-transformed.

WL%: weight loss %; Δ: change in a measurement from week 1 to week 12; BMI: Body mass index; FFM: Fat free mass; FFMI: Fat free mass index; BF%: Body fat percentage; VFM: Visceral fat mass; WC: Waist circumference; PDL1: Programmed death ligand 1; IL1-ra: Interleukin-1 receptor antagonist; CCL2: Chemokine ligand 2; CCL4: Chemokine ligand 4; CD40L: Cluster of differentiation-40 ligand; IFNγ: Interferon gamma; IL8: Interleukin-8; IP-10: IFNγ-induced protein.

**Supplementary Table 3: Correlation matrix of changes in anthropometric measures and serum biomarkers on week 24 relative to baseline in response to alternate day fasting plus low carbohydrate diet intervention.**

|  | WL% | ΔBMI | ΔFFM | ΔFFMI | ΔBF% | ΔVFM | ΔWC | ΔPDL1 | ΔCD40L | ΔCCL2 | ΔCCL4 | ΔIL1ra | ΔIFNγ | ΔIP10 | ΔIL8 |
| --- | --- | --- | --- | --- | --- | --- | --- | --- | --- | --- | --- | --- | --- | --- | --- |
| WL% | — |  |  |  |  |  |  |  |  |  |  |  |  |  |  |
| ΔBMI | **0.958***** | — |  |  |  |  |  |  |  |  |  |  |  |  |  |
| ΔFFM | **0.458*** | **0.422*** | — |  |  |  |  |  |  |  |  |  |  |  |  |
| ΔFFMI | **0.466*** | **0.431*** | **0.997***** | — |  |  |  |  |  |  |  |  |  |  |  |
| ΔBF% | **0.907***** | **0.912***** | 0.292 | 0.293 | — |  |  |  |  |  |  |  |  |  |  |
| ΔVFM | 0.263 | 0.26 | -0.214 | -0.193 | 0.281 | — |  |  |  |  |  |  |  |  |  |
| ΔWC | **0.496**** | **0.534**** | 0.024 | 0.03 | **0.456*** | 0.082 | — |  |  |  |  |  |  |  |  |
| ΔPDL1 | -0.04 | -0.064 | -0.229 | -0.226 | -0.175 | 0.174 | 0.044 | — |  |  |  |  |  |  |  |
| ΔCD40L | 0.2 | 0.168 | -0.137 | -0.156 | 0.243 | 0.157 | 0.265 | **0.525**** | — |  |  |  |  |  |  |
| ΔCCL2 | -0.152 | -0.244 | -0.048 | -0.059 | -0.257 | 0.396 | -0.233 | 0.176 | 0.149 | — |  |  |  |  |  |
| ΔCCL4 | 0 | 0.032 | -0.09 | -0.112 | 0.001 | 0.378 | 0.019 | **0.392*** | **0.406*** | 0.349 | — |  |  |  |  |
| ΔIL1ra | -0.014 | 0.035 | -0.21 | -0.209 | 0.019 | 0.098 | 0.236 | **0.409*** | 0.365 | 0.056 | **0.613***** | — |  |  |  |
| ΔIFNγ | -0.208 | -0.218 | -0.082 | -0.066 | -0.371 | 0.031 | -0.053 | **0.764***** | 0.302 | **0.393*** | 0.26 | 0.374 | — |  |  |
| ΔIP10 | 0.158 | 0.074 | -0.158 | -0.155 | 0.086 | 0.204 | 0.161 | **0.5*** | 0.039 | 0.044 | 0.164 | 0.169 | 0.306 | — |  |
| ΔIL8 | -0.075 | -0.042 | 0.108 | 0.098 | -0.188 | 0.183 | -0.114 | -0.033 | 0.013 | 0.262 | **0.573**** | 0.306 | -0.001 | -0.034 | — |

Data are presented as Pearson’s correlation coefficient.

Statistical significance is indicated by * p<0.05, ** p<0.01, or *** p<0.001.

Serum concentration of IP10, IL1ra, IFNγ, CCL4, and IL8 are log-transformed.

WL%: weight loss %; Δ: change in a measurement from week 1 to week 24; BMI: Body mass index; FFM: Fat free mass; FFMI: Fat free mass index; BF%: Body fat percentage; VFM: Visceral fat mass; WC: Waist circumference; PDL1: Programmed death ligand 1; IL1-ra: Interleukin-1 receptor antagonist; CCL2: Chemokine ligand 2; CCL4: Chemokine ligand 4; CD40L: Cluster of differentiation-40 ligand; IFNγ: Interferon gamma; IL8: Interleukin-8; IP-10: IFNγ-induced protein.
